# Higher Sensitivity of Soil Microbial Network Than Community Structure under Acid Rain

**DOI:** 10.3390/microorganisms9010118

**Published:** 2021-01-06

**Authors:** Ziqiang Liu, Hui Wei, Jiaen Zhang, Muhammad Saleem, Yanan He, Jiawen Zhong, Rui Ma

**Affiliations:** 1 Guangdong Provincial Key Laboratory of Eco-circular Agriculture, South China Agricultural University, Guangzhou 510642, China; liuziqiang0201@163.com (Z.L.); yananhe94@163.com (Y.H.); zhongjiawen@stu.scau.edu.cn (J.Z.); lmzhr2326@163.com (R.M.); 2 Department of Ecology, College of Natural Resources and Environment, South China Agricultural University, Guangzhou 510642, China; 3 Guangdong Engineering Technology Research Centre of Modern Eco-agriculture and Circular Agriculture, Guangzhou 510642, China; 4 Key Laboratory of Agro-Environment in the Tropics, Ministry of Agriculture and Rural Affairs, South China Agricultural University, Guangzhou 510642, China; 5 Guangdong Laboratory for Lingnan Modern Agriculture, Guangzhou 510642, China; 6 Department of Biological Sciences, Alabama State University, Montgomery, AL 36104, USA; msaleem@alasu.edu

**Keywords:** acid deposition, functional genes, high-throughput sequencing, microbial co-occurrence networks, N cycle

## Abstract

Acid rain (AR), as a global environmental threat, has profoundly adverse effects on natural soil ecosystems. Microorganisms involved in the nitrogen (N) cycle regulate the global N balance and climate stabilization, but little is known whether and how AR influences the structure and complexity of these microbial communities. Herein, we conducted an intact soil core experiment by manipulating the acidity of simulated rain (pH 7.5 (control, CK) vs. pH 4.0 (AR)) in subtropical agricultural soil, to reveal the differences in the structure and complexity of soil nitrifying and denitrifying microbiota using Illumina amplicon sequencing of functional genes (*amoA*, *nirS*, and *nosZ*). Networks of ammonia-oxidizing archaea (AOA) and *nirS*-carrying denitrifiers in AR treatment were less complex with fewer nodes and lower connectivity, while network of *nosZ*-carrying denitrifiers in AR treatment had higher complexity and connectivity relative to CK. Supporting this, AR reduced the abundance of keystone taxa in networks of AOA and *nirS*-carrying denitrifiers, but increased the abundance of keystone taxa in *nosZ*-carrying denitrifiers network. However, AR did not alter the community structure of AOA, ammonia-oxidizing bacteria (AOB), *nirS*-, and *nosZ*-carrying denitrifiers. Moreover, AR did not change soil N_2_O emissions during the experimental period. AOB community structure significantly correlated with content of soil available phosphorus (P), while the community structures of *nirS*- and *nosZ*-carrying denitrifiers both correlated with soil pH and available P content. Soil N_2_O emission was mainly driven by the *nirS*-carrying denitrifiers. Our results present new perspective on the impacts of AR on soil N-cycle microbial network complexity and keystone taxa in the context of global changes.

## 1. Introduction

Increasing energy consumption induced by continuing industrialization and urbanization has caused severe global environmental problems [1]. Of them, acid rain has become a significant environmental threat, which can profoundly influence global biogeochemical cycles and human health [1,2,3,4]. The anthropogenic emission of SO_2_ and NO_X_ is the main contributor to the acid rain [5]. In North America and Europe, SO_2_ emission has been reducing since 1990 due to many international joint control measures, while NO_X_ emission is still rising as a result of the increasing number of motor vehicles [6,7]. In contrast, in Asia, with the acceleration of industrialization and urbanization, both SO_2_ and NO_X_ emissions exhibit a continuous upward tendency [8,9,10,11]. Central and Southern China has received severe acid rain pollution since 1980s and become the third acid rain region after northeast America and central Europe across the globe [1,12]. Given that acid rain is predicted to continue in East Asia (e.g., China, Korea, and Japan) in the future [13], and microbe-driven biogeochemical functioning of ecosystem is sensitive to global changes [14,15]. Therefore, there is an urgent need to investigate the impacts of acid rain on ecosystem processes and functioning (e.g., soil N cycle) and involved microbial community.

Acid rain can affect soil N cycle, thereby influencing climate change [16]. In brushland and forestland, the N_2_O emissions could be stimulated by the acid rain (mainly SO_2_ deposition) [16], while contrasting responses of N_2_O emissions were observed in subtropical forestland soils [17]. However, limited studies clarified the responses of N_2_O emission to acid rain in the subtropical agricultural soil, which has been regarded as a considerable source of N_2_O emission [18]. Therefore, our understanding of underlying mechanisms of N_2_O emissions remains inadequate, and represents uncertainties in predicting the soil N-climate feedback under acid rain. Given the roles of microorganisms in regulating the exchanges of elements (e.g., C, N, and P) between the terrestrial and atmospheric pool, uncovering the responses of N-cycle microbial communities to acid rain is crucial for understanding the global N cycle. Previous studies have demonstrated that acid rain may affect soil microorganisms involved in N cycle by altering the soil properties and substrate availability. Of them, soil pH, as the key factor, profoundly influences the microbial abundance, community composition and activity [19,20]. Additionally, acid rain may alter soil substrate availability to ammonia oxidizers and denitrifiers through NO_3_^−^ leaching and plant N (e.g., dissolved organic N, NH_4_^+^, and NO_3_^−^) uptake from soil [21,22]. Generally, acid rain may negatively affect soil microbial communities by: (1) decreasing the soil pH, and increasing the exchangeable H^+^ and Al^3+^ [23], and (2) leaching the important base cations (e.g., K, Ca, Na, and Mg) [24,25]. Conversely, some studies reported that acid rain increased soil microbial diversity and richness [26], and stimulated microbial communities (e.g., bacteria) in short term [1], while some other studies found that acid rain did not influence the soil microorganisms [27]. Nevertheless, whether and how the N-cycle microbial communities respond to acid rain is still far from fully understood.

It is well established that soil N biogeochemical cycle includes nitrification and denitrification [21]. Of them, ammonia oxidation process (from NH_3_ to hydroxylamine, rate-limiting step of nitrification) is dominated by both ammonia-oxidizing archaea (AOA) and bacteria (AOB), and is marked by *amoA* gene [19]. Furthermore, in denitrification, nitrite-reducing denitrifiers that carry *nirK* and *nirS* genes participate in the N_2_O production, while nitrous oxide-reducing denitrifiers that carry *nosZ* gene control the N_2_O reduction [21]. However, most previous studies only focused on the microbial alpha- or beta-diversity, while the response of microbial co-occurrence relationship to acid rain is still unclear. Soil microbes do not exist in isolation but through mutualism, competition, or commensalism [28,29]. Microbial network dynamics are driven by extrinsic factors, such as host and plant community [30]. In addition, microbe–microbe interactions exert a considerable influence on microbiota structure and its ecological function, such as the resilience and resistance to external environmental stress [30]. Given that network analysis can unravel the complex microbe–microbe associations from their abundances across samples beyond the traditional analytical approaches, co-occurrence network analysis has become a widely applied technique to detect the potential associations among soil microbes [31]. In general, the microbiota can be divided into several different clusters/modules, which are internally highly interconnected. In addition, compared with environmental variables, microbial interactions may be more important in shaping community structure [32,33,34,35]. Yet, to our knowledge, whether and how acid rain affects the microbial co-occurrences networks in N cycle are still enigmatic.

In this study, we conducted an intact soil cores experiment to clarify the impacts of acid rain on the N-cycle microbial communities structure and complexity in ammonia oxidation and denitrification. We aimed to resolve the following questions: (1) Do N-cycle microbial co-occurrence network complexity and the abundance of keystone taxa change under acid rain? (2) Does acid rain alter the community structure of microbiota involved in ammonia oxidation and denitrification? (3) What are the main drivers of soil N_2_O emission under acid rain?

## 2. Materials and Methods

### 2.1. Collection of Soil Cores

Ten intact soil cores were taken from Zengcheng Teaching and Research Farm (113°38′ E, 23°14′ N) using polyvinyl chloride (PVC) tubes (60 cm of inside diameter, and 40 cm of depth) on July 2, 2017. This region, characterized by subtropical monsoon climate, experienced a mean annual temperature of 22 °C and mean annual precipitation of 1976.8 mm [36]. The soil in this region is lateritic red soil according to the Chinese soil taxonomic classification (Oxisols based on USDA soil taxonomy), and has been used for agricultural practices for several years [36]. The detailed field collection protocols and initial soil properties can be found in a previous study [37].

### 2.2. Experimental Design

After collection, all soil cores were transferred to a greenhouse (113°21′ E, 23°10′ N) of South China Agricultural University. After three months of equilibration [37], we applied two treatments to these soil cores: acid rain of pH 4.0 (AR) vs. non-acid addition (control, CK, local tap water, the average pH approximates to 7.5). Each treatment has five replicates. Acid rain solutions were prepared by mixing 0.5 mol L^−1^ sulfuric acids and 0.5 mol L^−1^ nitric acids at 2:1 mol concentration ratio [38], and then diluted by adding local tap water, and finally the pH values were calibrated to 4.0 using a portable Pro10 pH meter (YSI Inc./Xylem Inc., Yellow Springs, OH, USA). Throughout the experimental period, a total 623.1 mm acid rain was applied to each soil core according to the annual average acid precipitation in the past five years (2011–2015) in Guangdong [39,40]. To guarantee that each soil core is saturated by acid rain or tap water, 5 L of acid rain solution or tap water was accordingly sprayed into AR or CK soil core every five days with a sprayer since October 2017.

### 2.3. Measurement of N_2_O Flux

We collected N_2_O from each soil core twice a month from October 2017 to April 2018 using the static opaque chamber, and then determined the emissions by a gas chromatograph. Prior to gas collection, we removed all the plants within the PVC collars to avoid the interference by plants. After that, each cylindrical chamber equipped with an electric circulating fan was embedded into the groove of the PVC collar. Four 100 mL gas samples were taken from each sealed chamber with a syringe at 0, 10, 20, and 30 min, and accordingly injected into the empty aluminum foil bags. Then, 30 mL of gas sample was transferred from each bag into 15 mL pre-evacuated glass vials. Subsequently, the N_2_O concentrations were analyzed in a gas chromatograph. Finally, the N_2_O fluxes were calculated according to the established protocols [37].

### 2.4. Soil Sampling and Soil Physicochemical Properties Analysis

On April 11, 2018, five topsoil (0–10 cm, 2.5 cm of diameter) subsamples from each soil core were taken, and then mixed as a bulk sample. In total, 10 composite soil samples were obtained from all the soil cores. The samples were kept in an insulated box fitted with ice bags, and taken to the laboratory. Subsequently, all fresh soil samples were passed through a mesh screen (2 mm), and the rocks and plants residues were removed, and then each soil sample was divided into two parts. One part was air-dried in room temperature for quantifying soil chemical parameters (i.e., soil pH, available P, available K, alkaline N, NH_4_^+^-N, NO_3_^−^-N, and total C and N) following our parallel study [37], the other part was cryopreserved at −40 °C for further DNA extraction.

### 2.5. DNA Extraction, PCR Amplification, and Functional Genes Sequence Analysis

To identify and quantify the composition of microorganisms involved in N cycle, high-throughput sequencing of the AOA-*amoA*, AOB-*amoA*, *nirS,* and *nosZ* genes from all soil samples was conducted in Illumina MiSeq PE300 sequencing platform (Illumina Inc., CA, USA) with the assistances of Allwegene Technology Inc. (Beijing, China). The process of DNA extraction, PCR amplification, and functional genes sequencing were conducted according to the protocols described in Shi et al. [41]. The primers and reaction conditions of functional genes for PCR amplification in our study were presented in Table S1.

The obtained sequences were classified into different OTUs (operational taxonomical units) based on the 97% similarity level by UCLUST. Furthermore, taxonomic assignment and annotation of OTUs were achieved with NCBI taxonomy using BLASTN. Finally, we obtained OTUs feature tables for each functional gene. The raw sequencing data were deposited into the NCBI Sequence Read Archive (SRA) database with the accession No. PRJNA632992.

### 2.6. Network Inference, Construction, and Visualization

Microbial co-occurrence networks were inferred using CoNet v. 1.1.1 beta based on the OTUs tab-delimited file with taxonomy annotation [42]. In preprocessing and filtering menu, only OTUs that exist in all replicates of each treatment and had a sum relative abundance > 0.01% were retained for network analysis [31]. In the methods menu, Spearman correlation between all OTU pairs was performed, and the Spearman correlation (r) threshold was set to 0.7 [31]. After the two steps above, we obtained the initial networks that consist of all possible edges. Thus, we subsequently assessed the edge significance to discard invalid edges by launching the computation of permutation and bootstrap distributions with 1000 iterations, respectively, in the “Randomization” menu. To compute edge- and measure-specific permutation distributions, we selected the “edgeScores” routine along with “shuffle_rows” as the resampling method, and enabled “Renormalize” to alleviate the compositionality bias by a renormalization step [29]. To compute bootstrap distributions, we changed the “shuffle_rows” and “Renormalize” options to “bootstrap” and “Filter unstable edges”, respectively. In addition, we accordingly selected “brown” and “Benjamini-Hochberg” as the *p*-value merging method and multiple testing correction method, and set the *p*-value threshold to 0.05 [31]. Moreover, the permutation file generated from the previous step was loaded into CoNet as null distributions. Finally, only edges that passed the above two processes were remained. The nodes in the network correspond to different OTUs, while the edges correspond to significant correlations among OTUs. Moreover, the relationships between microbial taxa and soil factors were inferred in CoNet by importing the soil physicochemical properties via the “Metadata and features” submenu. “Transpose” and “Match samples” in “Features” option were selected to match soil physicochemical properties data to the OTUs tab-delimited data. Soil physicochemical properties were excluded from normalization steps [43]. 

Microbial co-occurrence networks were visualized using the Cytoscape v. 3.7.2 (https://cytoscape.org/). The “NetworkAnalyzer” tool was used to calculate network topology parameters (i.e., clustering coefficient, network diameter, shortest path, the average number of neighbors, and graph density). The OTUs own the highest degree and closeness centrality, but the lowest betweenness centrality was regarded as the keystone taxa [44]. In the network of AOA, the OTUs (degree = 5, closeness centrality = 1, and betweenness centrality = 0) were considered as the keystone taxa. In the network of *nirS*-carrying denitrifiers, the OTUs (degree > 10, closeness centrality > 0.354, and betweenness centrality < 0.103) were considered as the keystone taxa. In the network of *nosZ*-carrying denitrifiers, the OTUs (degree > 10, closeness centrality > 0.311, and betweenness centrality < 0.096) were considered as the keystone taxa. The co-occurrence networks of AOB were very small (< 10 nodes) and excluded in co-occurrence networks analysis.

### 2.7. Statistical Analysis

The alpha diversity indices (i.e., richness and evenness indices) of each functional gene were calculated in R v. 3.5.3 [45]. To compare soil physicochemical properties, and alpha diversity indices between acid rain and non-acid rain addition treatments, we used independent-samples *t*-test using SPSS 25.0 (IBM Corp., Armonk, NY, USA). Repeated-measures analysis of variances (ANOVA) was used to compare N_2_O fluxes among treatments. In addition, Pearson’s correlation analysis was performed to determine the relationships between the alpha diversity indices and soil physicochemical properties. Histogram and line chart were made with Origin 8.0 (OriginLab Corporation, Northampton, MA, USA).

The principle coordinate analysis (PCoA) based on the Bray–Curtis distance matrices was used to present the changes in the microbial community structure of ammonia oxidizers and denitrifiers among different treatments. Furthermore, we used permutational multivariate analysis of variance (PERMANOVA) to detect the differences in overall microbial community structure across treatments. The Mantel test based on the Spearman’s correlations was performed to explore the relationships between functional community composition and soil physicochemical property. The PCoA, PERMANOVA, and Mantel test were conducted using “vegan” package in R.

Structural equation modeling (SEM) was conducted in AMOS 21.0 (SPSS Inc., Chicago, IL, USA) to determine if and how soil physicochemical properties and N-cycle microorganisms influenced the N_2_O flux rates. Before modeling, the normal distribution of data was checked. The observed number of OTUs in each functional gene was used in SEM and was log-transformed. The goodness of model fits was based on the chi-square test (*p* > 0.05), comparative fit index (CFI > 0.95), and root mean square errors of approximation (RMSEA < 0.05) [46]. Only significant pathways (*p* < 0.05) were shown in the model.

## 3. Results

### 3.1. The AR Effects on Microbial Co-occurrence Networks

**Figure 1:**
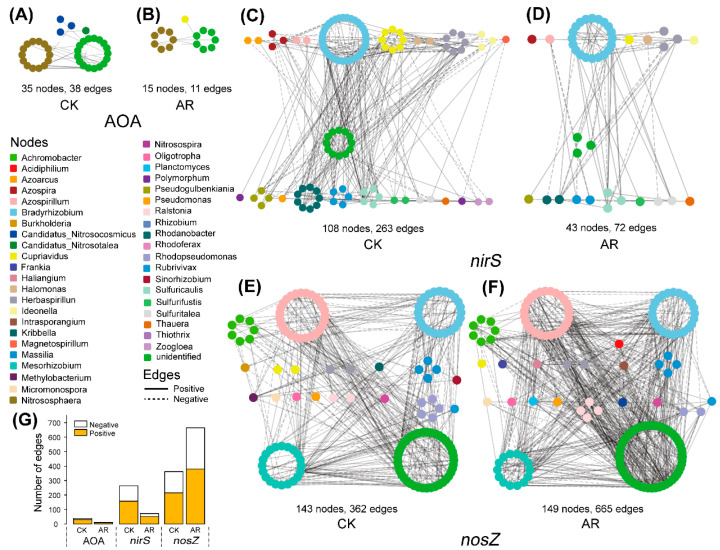
Co-occurrence networks of the microbial communities in control (CK) and acid rain (AR) treatments. Nodes represent different OTUs, and OTUs are colored by genus-level taxonomy. Edges indicate the significant (*p* < 0.05) relationships among different OTUs. AOA: ammonia-oxidizing archaea (**A**,**B**); *nirS* and *nosZ*: denitrifiers carrying *nirS* and *nosZ* genes, respectively (**C**–**F**). Number of edges (**G**). CK: non-acid addition, AR: pH 4.0 acid rain.

The network structure and topology under AR treatment were remarkably different from that of CK (Figure 1, Table 1). The networks of AOA and *nirS*-carrying denitrifiers in AR treatment were less connected and complex than that of CK based on the number of nodes and edges, and the average number of neighbors (Figure 1A–D, Table 1). In contrast, the network of *nosZ*-carrying denitrifiers in the AR treatment had higher complexity and connectivity relative to the CK (Figure 1E,F, Table 1). The differences in complexity and connectivity of networks could be ascribed to the abundance of keystone N-cycle taxa. In the AOA network, the CK harbored six keystone taxa (OTUs) that belonged to the genus *Nitrososphaera*, whereas none of these was detected in the AR treatment. Similarly, in the *nirS*-carrying denitrifiers network, the CK harbored five keystone taxa (OTUs) that belonged to the genus *Bradyrhizobium*, *Cupriavidus*, *Rhodanobacter*, *Bradyrhizobium,* and *Azospira*, while none of these was observed in the AR treatment. However, in *nosZ*-carrying denitrifiers network, the AR treatment harbored 37 keystone taxa (OTUs), while only 5 in the CK. These keystone taxa in the AR treatment belonged to the genus *Azospirillum*, *Bradyrhizobium*, *Mesorhizobium*, *Achromobacter*, *Rhodopseudomonas*, *Haliangium*, *Intrasporangium*, *Ralstonia,* and *Rhizobium*, while the keystone taxa in the CK belonged to the genus *Methylobacterium* and *Bradyrhizobium*. In addition, taxa were prone to co-occur (solid edges) rather than co–exclude (dashed edges) with 57–79% positive associations existing in the AOA, *nirS*-, and *nosZ*-carrying denitrifiers (Figure 1G).

**Figure 2:**
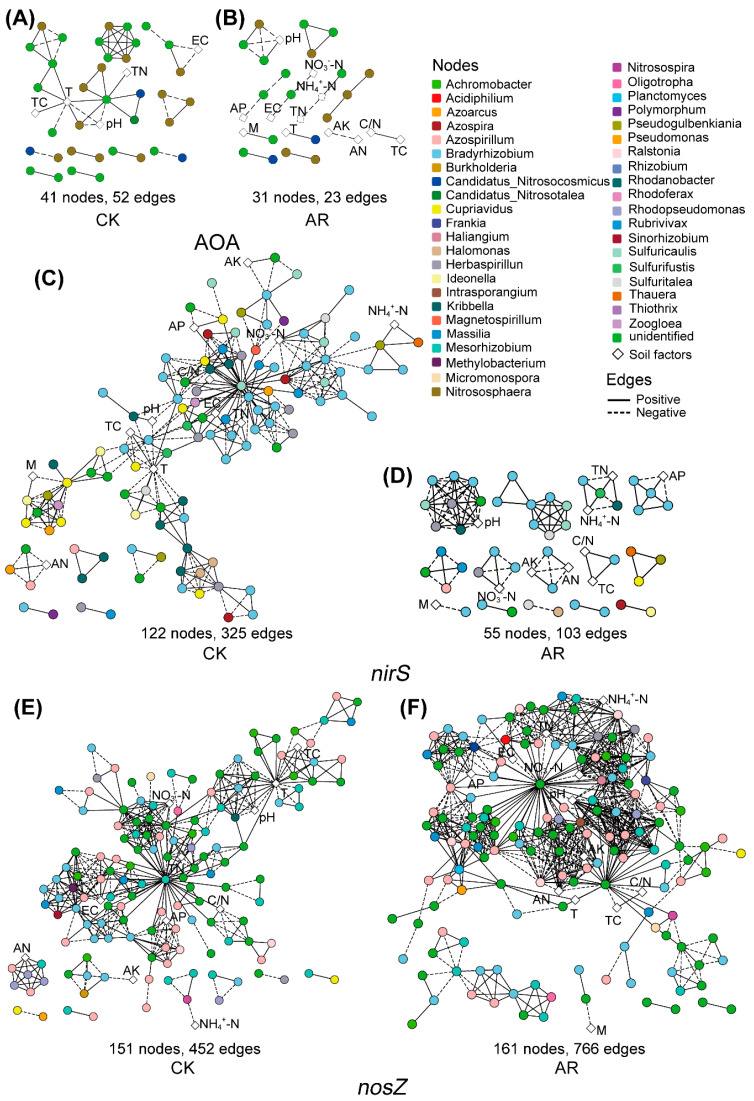
Co-occurrence networks of microbial communities and soil chemical factors in CK and AR treatments. AP: available P; AK: available P; AN: alkaline N; NH_4_^+^-N: ammonium-N; NO_3_^−^-N: nitrate-N; TC: total C; TN: total N; C/N: the ratio of total C to total N; M: soil moisture; EC: soil electrical conductivity; T: soil temperature. Nodes represent OTUs and soil factors with different shapes, and OTUs are colored by genus-level taxonomy. Edges indicate the significant (*p* < 0.05) relationships among different OTUs. AOA: ammonia-oxidizing archaea (**A**,**B**); *nirS* and *nosZ*: denitrifiers carrying *nirS* and *nosZ* genes, respectively (**C**–**F**). CK: non-acid addition, AR: pH 4.0 acid rain.

Similar to the microbial co-occurrence network structure, the networks of AOA, *nirS*-carrying denitrifiers and associated soil factors in the AR treatment were smaller with less nodes and edges compared to the CK (Figure 2A–D), while the network of *nosZ*-carrying denitrifiers and soil factors in the AR treatment has higher complexity and connectivity relative to the CK (Figure 2E,F). In addition, the relationships between microbial taxa and soil pH in the AR treatment differed from the CK. Specifically, in AOA networks, the soil pH was negatively correlated with all the microbial taxa (e.g., genus *Nitrososphaera*) in the CK, while it had positive connectivity to an OTU belonged to the phylum Thaumarchaeota in the AR treatment (Figure 2A,B). In the *nirS*-carrying denitrifiers network, the soil pH was only positively correlated to an OUT belonging to genus *Rhodanobacter* in CK, while it positively correlated to the OTUs belonging to genus *Bradyrhizobium* and *Herbaspirillum* in the AR treatment (Figure 2C,D). In the *nosZ*-carrying denitrifiers network, only an OTU belonged to the genus *Bradyrhizobium* and was positively related to soil pH in the CK, while OTUs belonging to genus *Azospirillum*, *Bradyrhizobium*, *Mesorhizobium,* and *Rhodopseudomonas* were positively related to soil pH in the AR treatment (Figure 2E,F, Figure S1A,B).

### 3.2. The AR Effects on Microbial Diversity, Community Structure, and N_2_O Emissions

The AR treatment decreased the number of OTUs and Chao1 richness index of the AOA-*amoA* gene (*p* < 0.05, Figure S2A,B), while it did not change the alpha diversity indices of AOB-*amoA*, *nirS,* and *nosZ* genes in the experimental soil (Figure S2A–F). The PCoA analysis showed that the microbial community composition of ammonia oxidizers (AOA and AOB) and *nirS*- and *nosZ*-carrying denitrifiers did not change under AR treatments (Figure S3). In addition, AR treatment did not alter soil N_2_O fluxes during the experimental period (Figure S4).

**Figure 3:**
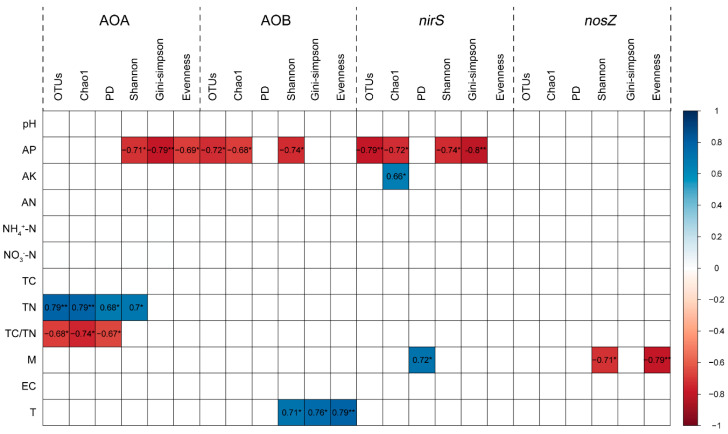
Pearson correlations between alpha diversity indices of functional genes and soil physicochemical properties. AP: available P; AK: available K; AN: alkaline N; NH_4_^+^-N: ammonium-N; NO_3_^−^-N: nitrate-N; TC: total C; TN: total N; M: soil moisture; EC: soil electrical conductivity; T: soil temperature. OTUs: observed number of OTUs; Chao1: Chao1 richness index; PD: phylogenetic diversity index; Shannon: Shannon diversity index; Gini–Simpson: Gini–Simpson index; Evenness: Shannon’s evenness index. Cell entries are Pearson correlation coefficients (r) between two indices. Only significant correlations are shown. * *p* < 0.05, ** *p* < 0.01.

For AOA, the number of OTUs, Chao1, phylogenetic, and Shannon diversity indices positively correlated with the soil total N content, while the Shannon diversity, Gini–Simpson and Shannon’s evenness indices were negatively related to the soil available P content (*p* < 0.05, Figure 3). For AOB, the OTUs number, Chao1, and Shannon diversity indices were negatively related to soil available P content, while Shannon diversity, Gini–Simpson, and Shannon’s evenness indices positively correlated with the soil temperature (*p* < 0.05, Figure 3). For *nirS*-carrying denitrifiers, the OTUs number, Chao1, Shannon diversity and Gini–Simpson indices were negatively related to the soil available P content, while phylogenetic diversity index was positively related to the soil moisture (*p* < 0.05, Figure 3). However, in *nosZ*-carrying denitrifiers, we only observed significantly negative relationships of Shannon diversity, and Shannon’s evenness indices with the soil moisture (*p* < 0.05, Figure 3).

**Figure 4:**
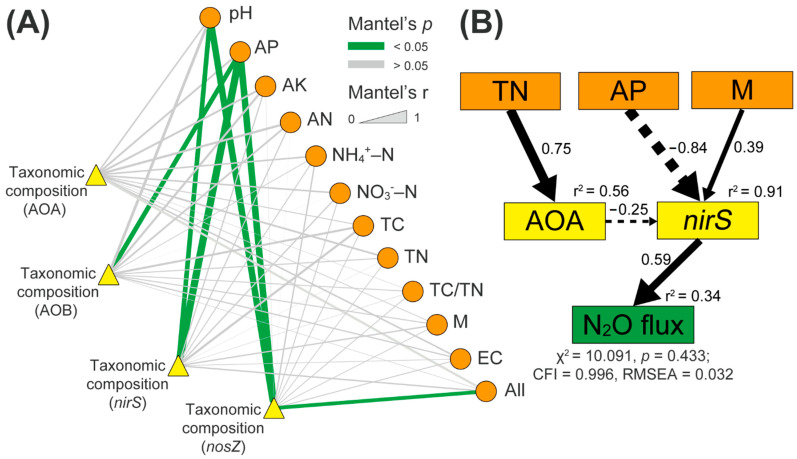
(**A**) Mantel test of relationships between the microbial community composition of AOA, ammonia-oxidizing bacteria (AOB), *nirS*-, and *nosZ*-carrying denitrifiers, and soil properties. Edge width is linked to the Mantel’s r statistic. AP: available P; AK: available K; AN: alkaline N; NH_4_^+^-N: ammonium-N; NO_3_^−^-N: nitrate-N; TC: total C; TN: total N; M: soil moisture; EC: soil electrical conductivity; All: all soil properties. AOA, AOB, *nirS*, and *nosZ*: ammonia-oxidizing archaea, bacteria, *nirS-,* and *nosZ*-carrying denitrifiers, respectively. (**B**) Structural equation model (SEM) analysis showed how soil factors and N-cycle microbial abundance affect soil N_2_O flux rates. AOA, *nirS*: the OTUs values of ammonia-oxidizing archaea, and *nirS*-carrying denitrifiers, respectively. Results of model fitting: chi-square = 10.091, *p* = 0.433 > 0.05, comparative fit index (CFI) = 0.996 > 0.95, root mean square errors of approximation (RMSEA) = 0.032 < 0.05. Solid and dashed arrows denote significant positive and negative effects (*p* < 0.05), respectively. The r^2^ values next to each parameter represent the variance explained by other parameters. The values upon each arrow indicate the standardized path coefficients.

The links between N-cycle microbial community composition and soil properties were detected using the Mantel test (Figure 4A). AOB community composition was significantly correlated with soil available P content (Spearman’s coefficient r = 0.424, *p* < 0.05, Figure 4A). Both soil pH and available P content were correlated to the microbial community composition of *nirS*- and *nosZ*-carrying denitrifiers (*p* < 0.05, Figure 4A). However, no significant relationships among AOA community composition and soil factors were observed (Figure 4A).

The SEM analyses indicated that soil total N explained 56% of the total variance of the abundance of AOA (Figure 4B). The observed changes in soil moisture, available P content, and abundance of AOA explained 91% of the total variance of the abundance of *nirS*-carrying denitrifiers (Figure 4B). In total, 34% of the total variances in the soil N_2_O emissions were explained by the change in the abundance of *nirS*-carrying denitrifiers (Figure 4B).

## 4. Discussion

### 4.1. Impact of Acid Rain on Microbial Co-occurrence Patterns of AOA, nirS-, and nosZ-carrying Denitrifiers

Both AOA and *nirS*-carrying denitrifiers harbor a less complex network with fewer nodes, while *nosZ*-carrying denitrifiers develop a more complex network with more highly connected nodes under acid rain condition. The highly connected N-cycle keystone taxa play crucial roles in the community composition and functions of soil microbiota, and its ecological complexity is mostly determined by the number of associations that its members share [47]. In our study, acid rain reduced the abundance of keystone OTUs in the networks of AOA and *nirS*-carrying denitrifiers, while it increased the abundance of keystone OTUs in the networks of *nosZ*-carrying denitrifiers. Thus, the distinct networks complexity of AOA, *nirS*-, and *nosZ*-carrying denitrifiers under acid rain condition could be ascribed to the different abundance of N-cycle keystone taxa in their networks. Although previous studies have reported changes of keystone taxa abundance in microbial networks under various environmental conditions [47,48,49], how the keystone taxa involved in the N cycle respond to acid rain remains scant [50]. Therefore, an important question is how acid rain affects the keystone taxa of microbial co-occurrence network in the N cycle. Previous studies reported that climatic variables (e.g., annual average temperature and precipitation and atmospheric CO_2_ concentrations) [31,51], and edaphic variables (e.g., pH, organic carbon, and available Ca and Mg) [31,52] had a strong impact on microbial networks and topological features. Among these, soil pH has been considered as the critical control on the structure and complexity of microbial communities [31,53]. In addition, a recent study reported that the keystone taxa in agroecosystems were affected by the soil pH and phosphorus levels, which drive the reassembly and development of microbes in the soil [47]. Liang et al. [54] found that the network topological parameters (e.g., connectivity, module numbers, and modularity) were all decreased with increasing the soil acidity (from 6.2 to 5.8) by the oil contamination. Moreover, Nielsen et al. [55] found that biochar additions could increase the associations between microbial taxa in network due to an increased soil pH (from 4.6 to 4.8). Thus, we inferred that acid rain-induced soil acidification changed the number of keystone taxa in the microbial co-occurrence networks. However, there may exist different pH threshold points of balance between the inhibitory effect of soil acidification and acid rain-induced positive “fertilization effect” (NO_3_^-^ input by acid rain) in AOA, *nirS*-, and *nosZ*-carrying denitrifiers. Based on these relationships, when the inhibitory effect of soil acidification exceeds the acid rain-induced positive “fertilization effect” on microbial communities, acid rain reduced the abundance of keystone taxa in AOA and *nirS*-carrying denitrifiers networks. Although the soil pH significantly decreased in our study (from 5.1 to 4.7, Table S2), the abundance of keystone taxa in *nosZ*-carrying denitrifiers networks increased rather than decreased as we expected. Thus, acid rain-induced positive “fertilization effect” may exceed the adverse impacts of soil acidification on microbial communities, as the soil available P, ammonium-N, and nitrate-N tended to increase under acid rain exposure (Table S2).

The microbial interactions along with the presence and absence of microbial taxa, may also determine the intensity of microbial-driven ecosystem functions [32,56,57]. Microbial interactions could influence ecosystem functioning directly by regulating processes of energy and material flow, or indirectly by altering the abundances or traits of keystone taxa [56]. The greater number of interactions among microbial groups usually corresponds to the higher level of cooperation, and exchange events within microbiota [51,57]. Moreover, more complex networks are usually more stable under various external environmental stress [58]. In our study, positive co-occurrence associations prevailed among different microbial taxa (Figure 1G), resulting in positive feedback and co-oscillation [59]. However, acid rain reduced the positive connections in AOA and *nirS*-carrying denitrifiers networks, while it increased the positive connections in *nosZ*-carrying denitrifiers network. In this line, the acid rain may reduce the resistance or resilience of AOA and *nirS*-carrying denitrifiers to external stress, while it may increase the resistance or resilience of *nosZ*-carrying denitrifiers networks due to a greater functional redundancy. However, further empirical studies are necessary to complement and corroborate these observations.

### 4.2. Impact of Acid Rain on Microbial Diversity, Community Structure, and N_2_O Emissions

Acid rain decreased the OTUs and Chao1 of AOA, while it did not change the alpha diversity indices of AOB, *nirS*-, and *nosZ*-carrying denitrifiers. This could be attributed to the distinct sensitivities of AOA, AOB, *nirS*-, and *nosZ*-carrying denitrifiers to the acid rain. Previous studies reported that AOA were more vulnerable than AOB to long-term fertilization treatments in the acidic soils [60,61]. Additionally, Li et al. [50] reported that acid rain reduced the abundance of AOA-*amoA*, while it had no effect on the abundances of AOB-*amoA*, *nirS,* and *nosZ* in lateritic red soil (the same soil type used in our study). Thus, we speculated that AOB, *nirS*-, and *nosZ*-carrying denitrifiers showed a higher resilience to acid rain, as compared with that of AOA. In addition, we found that the alpha diversity indices of AOA were mainly related to soil total N content and C/N ratio, while the diversity indices of AOB, *nirS*-carrying denitrifiers were mainly related to the soil available P content (Figure 3). Meanwhile, in our study, acid rain addition changed soil total N content and C/N ratio, while it did not alter soil available P content (Table S2). Therefore, observed distinct sensitivity of AOA, AOB, *nirS*-, and *nosZ*-carrying denitrifiers to acid rain could be ascribed to their different nutritional requirements.

Acid rain did not alter the microbial community structure of AOA, AOB, *nirS*-, and *nosZ*-carrying denitrifiers though the soil pH decreased (from 5.1 to 4.7), which was in line with the findings of a recent trial conducted in temperate and subtropical forest soils [62]. Previous studies found that environmental factors (e.g., soil pH and C and N availability) directly or indirectly determined soil microbial communities [63,64,65,66,67], among these, the soil pH was the strongest driver of microbial composition [65]. Tang et al. [68] demonstrated that N additions altered the community compositions of AOA when soil pH declined (from 4.6 to 4.3) after N applications in Chinese fir plantation. However, although N additions significantly decreased the soil pH in the temperate (from 5.3 to 4.9) and subtropical forest (from 3.9 to 3.8), it did not change the community compositions of AOA, AOB, and *nirS*-denitrifier [62]. It is very likely that microbial communities are resistant to the external environmental changes, and there may be different ecosystem-specific thresholds of soil pH beneath which the changes in soil pH will not alter the soil microbial communities. In addition, microbial composition might be resilient and maintain their inherent structure by increasing the investment of C source once encountered external stress such as changes in moisture, temperature, pH, and salinity [69]. Furthermore, the results from Mantel test showed that the alterations in the community structure of AOB, *nirS*-, and *nosZ*-carrying denitrifiers were all highly related to changes in soil available P content, which was consistent with a previous study [70]. Thus, another possible interpretation for the unaltered community composition of AOA, AOB, *nirS*-, and *nosZ*-carrying denitrifiers could be the unchanged contents of soil nutrients (e.g., soil available P) under acid rain in our study (Table S2).

Acid rain did not alter the soil N_2_O emission during the investigation period of our study (Figure S4), which was consistent with a previous study [18]. Previous studies reported that acid rain had promoted, inhibited or had no influence on soil N_2_O emissions, mainly depending on the initial soil pH and other soil parameters such as available C and N, microbial community structure and activity [17,71,72,73,74,75]. In our study, soil N_2_O flux was directly driven by the microbial abundance of *nirS*-carrying denitrifiers (Figure 4B), which was in line with previous reports [76,77]. Meanwhile, the abundance of *nirS*-carrying denitrifiers was not changed though the network associations among *nirS*-carrying denitrifiers decreased under the acid rain exposure (Figure S2, Figure 1D). In addition, soil N_2_O flux was highly correlated with soil NO_3_^−^-N contents in our parallel study [37]. Therefore, the unaltered N_2_O emissions could be attributed to the indistinctive differences in the abundance of denitrifiers, and soil chemical properties (especially soil NO_3_^−^-N contents) under acid rain condition.

### 4.3. Idiosyncratic Responses of Microbial Co-occurrence Network, Community Structure, and Function to Acid Rain

There are widely accepted hypotheses in which microbial composition might not affect ecosystem functioning under disturbances [78]. Microbial community can be resistant (e.g., due to redundancy among microbial species) or resilient (i.e., composition changes but quickly return to its original state) to external changes [78]. Even if microbial composition changes, communities may continue to support ecosystem processes due to functional redundancy, in which the new community might be functionally similar to the original (i.e., new microbial communities come to fill the ecological roles of original microorganisms), such that the process is maintained even after some taxa are lost [78]. In our study, we found that acid rain changed microbial network structure and the abundance of N-cycle keystone taxa, while it did not alter soil N_2_O emissions (i.e., function). This indicates that the microbial network relationships could be highly sensitive to the environmental changes than the microbial community composition and functions. In addition, it is possible that the differences in network associations are early indications of changes in the microbial community structure. Therefore, once acid rain continues, and the acidification exceeds the pH thresholds of the soil, it may change the soil microbial network relationships, community structure, diversity, and functions (e.g., N_2_O emissions).

However, since recent studies have suggested that taxa in microbial communities may not be functionally similar [78], we considered that the alleged “functional redundancy” is a carbon investment tradeoffs strategy among high growth yield, resource acquisition and stress tolerance, rather than inherent characteristics of microbial communities against external environmental disturbance. Previous studies confirmed that microorganisms had a carbon allocation tradeoff among cell growth and stress tolerance, which ultimately could influence microbial role in the nutrient cycle [69,79]. Therefore, it is possible that carbon and energy could be preferentially invested to maintain the metabolic activities of microbial functional groups, which was the potential interpretation why microbial functions did not change even after alterations in microbial associations. However, once the supply of external carbon/nitrogen source is insufficient, both the functional and nonfunctional microbes will be affected, and ultimately having consequences on ecosystem functions. Therefore, the relationship between microbial community structure and function will be mainly dependent on the supply of external carbon/nitrogen sources. In our study, acid rain addition reduced soil total N, while it still did not change soil N_2_O emissions. Thus, we inferred that there existed a threshold of carbon/nitrogen, beneath which the existing available nutrients in the soil were insufficient to support the metabolic activities of the microbial functional groups, which ultimately led to changes in microbial functions at some level (e.g., N_2_O emissions).

**Figure 5:**
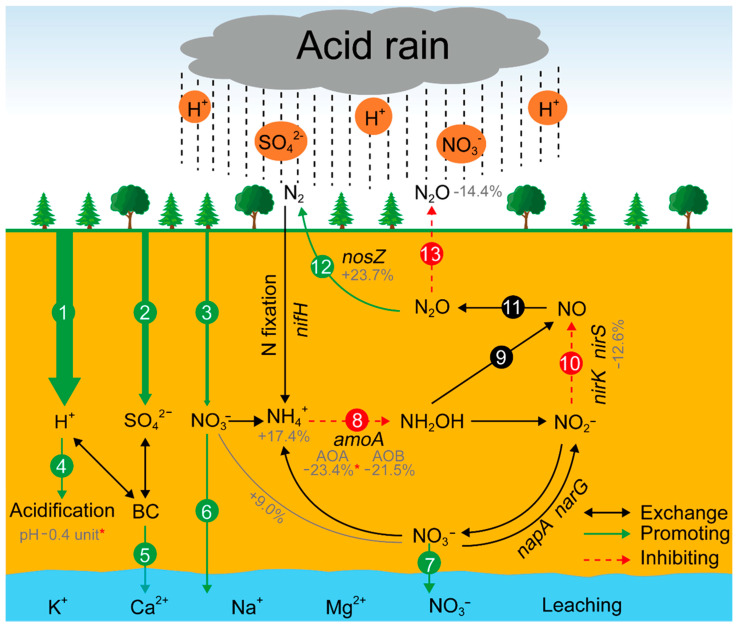
Predicted model showing the changes in N_2_O flux under continuing acid rain based on the previous established N cycle paths [21]. (1), (2), and (3) H^+^, SO_4_^2−^, and NO_3_^−^ inputs carried by acid rain; (4) soil acidification by H^+^ input; (5) BC (base cations) leaching; (6) and (7) NO_3_^-^ leaching; (8) ammonia oxidation (from NH_3_ to hydroxylamine); (9) nitrifier denitrification; (10) nitrite reduction; (11) NO reduction; (12) the reduction of N_2_O to N_2_; and (13) soil N_2_O emissions. The percentages under soil nutrients and functional genes correspond to the changes in soil parameters and OTUs number in our study. * *p* < 0.05. Two-way arrows indicate the ions exchange. Green and red arrows represent the predicted positive and negative changes with continuous acid rain input, respectively.

Overall, with continuous acid rain input (mainly H^+^) and no extra carbon/nitrogen inputs, the community structure and diversity of AOA, AOB, *nirS*-, and *nosZ*-carrying denitrifiers may change, and ultimately having a possible effect on soil N_2_O emissions. Firstly, the acid rain-induced soil acidification would change the microbial networks structure, community composition, and diversity of AOA and *nirS*-carrying denitrifiers. As a result, the functions of AOA and *nirS*-carrying denitrifiers would be restricted or altered. Therefore, the production of hydroxylamine (NH_2_OH) and NO may decrease as the paths of ammonia-oxidation and nitrite reduction are restricted (Figure 5, paths 8 and 10). Meanwhile, the decreased NH_2_OH may also lead to less production of NO from nitrifier denitrification (Figure 5, path 9). Consequently, the N_2_O production may decrease due to insufficient supply of NO for the process of NO reduction (Figure 5, path 11). Additionally, in our study, the microbial interactions and keystone taxa abundance in the *nosZ*-carrying denitrifiers increased, which indicated that the reduction of N_2_O to N_2_ would be stimulated with a continuous acid rain exposure (Figure 5, path 12). Ultimately, the soil N_2_O emissions may be decreased under acid rain with excess H^+^ input (Figure 5, path 13). However, these predictions must be complemented and corroborated with further experiments to uncover their true alterations under acid rain, which is what we are going to do in the future.

## 5. Conclusions

Acid rain simplified the networks of AOA and *nirS*-carrying denitrifiers, while increasing the complexity and connectivity of network of *nosZ*-carrying denitrifiers. Moreover, AR decreased the keystone taxa abundance in the networks of AOA and *nirS*-carrying denitrifiers, while it increased the keystone taxa abundance in the network of *nosZ*-carrying denitrifiers. Acid rain did not change the community structure of AOA, AOB, *nirS*-, and *nosZ*-carrying denitrifiers. Meanwhile, acid rain showed no obvious effects on soil N_2_O emissions during the experimental period.

## Figures and Tables

**Table 1:** Key topological indices of microbial networks of ammonia-oxidizing archaea (AOA), *nirS*-, and *nosZ*-carrying denitrifiers.

Microbial Community		Topological Features						
	Treatment	Nodes ^a^	Edges ^b^	Clustering Coefficient ^c^	Network Diameter ^d^	Shortest Paths ^e^	Avg. Number of Neighbors ^f^	Graph Density ^g^
AOA	CK	35	38	0.424	2	88	2.171	0.064
	AR	15	11	0.400	2	24	1.467	0.105
*nirS*	CK	108	263	0.723	10	5850	4.870	0.046
	AR	43	72	0.756	2	164	3.349	0.080
*nosZ*	CK	143	362	0.773	7	8756	5.063	0.036
	AR	149	665	0.701	9	16248	8.926	0.060

^a^ Operational taxonomical units (OTUs) colored based on genus-level taxonomy; ^b^ Number of connections/correlations among nodes; ^c^ Denoting the highly interconnected structure of a particular region of a network; ^d^ The largest distance between two nodes; ^e^ Indicating how quickly the information can be transferred between two nodes; ^f^ Indicating the average connectivity of a node; ^g^ The ratio of the number of edges to the number of possible maximum edges. AOA: ammonia-oxidizing archaea; *nirS* and *nosZ*: *nirS-* and *nosZ*-carrying denitrifiers.

## Data Availability

The raw sequencing data were deposited into the NCBI Sequence Read Archive (SRA) database with the accession No. PRJNA632992. The data for analysis in this study are available on request from the corresponding author.

## Supplementary Materials

https://www.mdpi.com/2076-2607/9/1/118/s1, Table S1: Primers used for PCR amplification of archaeal and bacterial *amoA*, *nirS*, and *nosZ* genes sequences; Table S2: Soil chemical properties among treatments (mean ± SE, *n* = 5); Figure S1: Subnetwork of microbial taxa and soil pH in CK (A) and AR (B) treatments. Connections stand for Spearman’s correlation with r > 0.7 and statistically significant at *p* < 0.05. Nodes represent OTUs and soil factors with different shapes, and OTUs are colored by genus-level taxonomy. *nosZ*: denitrifiers harboring *nosZ* gene; Figure S2: The microbial community richness and diversity indices of functional genes among treatments. OTUs: observed number of OTUs (A); Chao1: Chao1 richness index (B); PD: phylogenetic diversity index (C); Shannon: Shannon diversity index (D); Gini–Simpson: Gini–Simpson index (E); Evenness: Shannon’s evenness index (F). CK: non-acid addition, AR: pH 4.0 acid rain. Data were presented as mean ± standard error (*n* = 5). ** *p* < 0.01, * *p* < 0.05; Figure S3: The principle coordinate analysis (PCoA) of the microbial communities structure of AOA (A), AOB (B), *nirS*-harboring denitrifiers (C) and *nosZ*-harboring denitrifiers (D). CK: non-acid addition, AR: acid rain of pH 4.0. Five points in the same color block represent one treatment. The *p*-values refer to PERMANOVA results; Figure S4: N_2_O fluxes over sampling time. CK: non-acid addition, AR: pH 4.0 acid rain. Data were presented as mean ± standard error (*n* = 5).
