# Supplementary material for: Higher Sensitivity of Soil Microbial Network Than Community Structure under Acid Rain
Source: Microorganisms. 2021 Jan 6;9(1):118. doi: 10.3390/microorganisms9010118 (PMC7825572; doi:10.3390/microorganisms9010118)
Supplement: Supplementary file 1 [file microorganisms-09-00118-s001.pdf]

Supplementary Materials

Table S1. Primers used for PCR amplification of archaeal and bacterial *amoA*, *nirS* and *nosZ* genes sequences.

Functional genes	Primer name	Sequence (5'-3')	PCR conditions	Reference
AOA-*amoA*	Arch-amoA26F	GACTACATMTTCTAYACWGAYTGGGC	94°C for 5 min × 1 cycle;	(Park et al., 2008)
	Arch-amoA417R	GGKGTCATRTATGGWGGYAAYGTTGG	94°C for 30 s, 53°C for 30 s, 72 °C for 60 s × 35 cycles;<br>72 °C for 7 min × 1 cycle	
AOB-*amoA*	amoA-1F	GGGGTTTCTACTGGTGGT	94°C for 5 min × 1 cycle;	(Rotthauwe et al., 1997)
	amoA-2R	CCCCTCKGSAAAGCCTTCTTC	94°C for 30 s, 50°C for 30 s, 72 °C for 60 s × 35 cycles;<br>72 °C for 7 min × 1 cycle	
nirS	Cd3aF	GTSAACGTSAAGGARACSGG	94°C for 5 min × 1 cycle;	(ThrobÃ Ck et al., 2004)
	R3cd	GASTTCGGRTGSGTCTTGA	94°C for 30 s, 57°C for 30 s, 72 °C for 60 s × 30 cycles;<br>72 °C for 7 min × 1 cycle	
nosZ	nosZ-F	CGYTGTTCMTCGACAGCCAG	94°C for 5 min × 1 cycle;	(Kloos et al., 2001)
	nosZ1622R	CGSACCTTSTTGCCSTYGCG	94°C for 60 s, 63°C for 30 s, 72 °C for 60 s × 35 cycles;<br>72 °C for 7 min × 1 cycle	

REFERENCES

Kloos, K., Mergel, A., Rösch, C., Bothe, H. (2001). Denitrification within the genus *Azospirillum* and other associative bacteria. *Australian Journal of Plant Physiology*. 28, 991-998

Park, S., Park, B., Rhee, S. (2008). Comparative analysis of archaeal 16S rRNA and *amoA* genes to estimate the abundance and diversity of ammonia-oxidizing archaea in marine sediments. *Extremophiles*. 12, 605-615

Rotthauwe, J.H., Witzel, K.P., Liesack, W. (1997). The ammonia monooxygenase structural gene *amoA* as a functional marker: Molecular fine-scale analysis of natural ammonia-oxidizing populations. *Appl. Environ. Microb.* 63, 4704-4712

ThrobÃ Ck, I.N., Enwall, K., Jarvis, Ã.S., Hallin, S. (2004). Reassessing PCR primers targeting *nirS*, *nirK* and *nosZ* genes for community surveys of denitrifying bacteria with DGGE. *FEMS Microbiol. Ecol.* 49, 401-417

Table S2. Soil chemical properties among treatments (mean ± SE, *n* = 5).

	pH	AP<br>mg kg^{-1}	AK<br>mg kg^{-1}	AN<br>mg kg^{-1}	NH_4^+-N<br>mg kg^{-1}	NO_3^--N<br>mg kg^{-1}	TC<br>g kg^{-1}	TN<br>g kg^{-1}	TC/TN
CK	5.11±0.07	5.3±1.3	51.3±7.2	62.9±5.6	4.70±0.96	4.91±0.74	11.2±0.1	2.10±0.03	5.31±0.04
AR	4.71±0.12	11.3±3.3	46.6±5.6	63.8±1.1	5.52±0.15	5.35±0.20	11.6±0.3	1.99±0.01	5.81±0.16
p-values	**0.021***	0.128	0.625	0.890	0.447	0.586	0.255	**0.004****	**0.030***

CK: control, AR: acid rain with pH of 4.0. AP: available phosphorus; AK: available potassium; AN: alkaline nitrogen; NH_4^+-N: ammonium nitrogen; NO_3^--N: nitrate nitrogen; TC: total carbon; TN: total nitrogen. The *p*-values refer to the independent-samples t-test results. ** Different at $p < 0.01$, * Different at $p < 0.05$, ^{ns} Non-different.

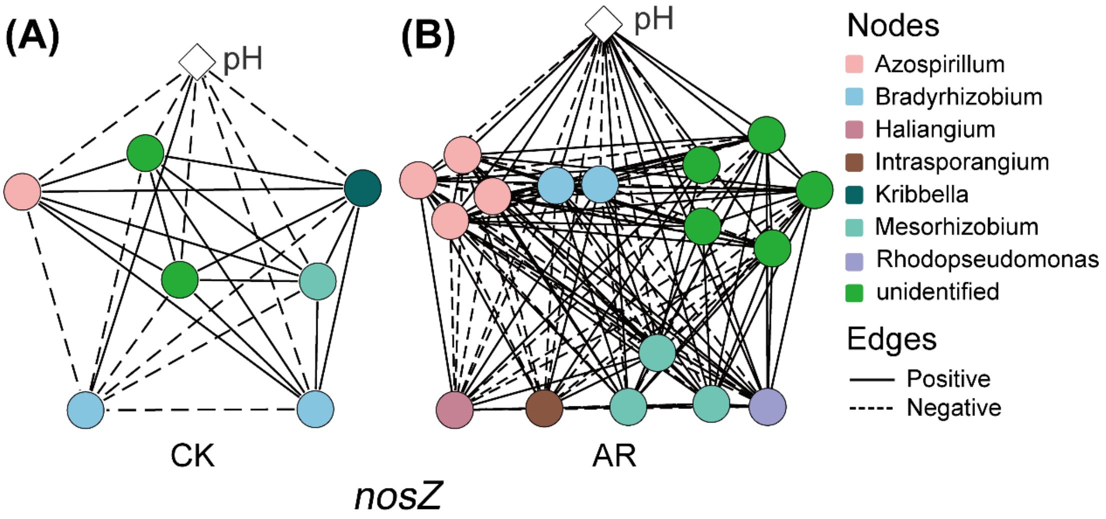

Figure S1. Subnetwork of microbial taxa and soil pH in CK **(A)** and AR **(B)** treatments. Connections stand for Spearman's correlation with r> 0.7 and statistically significant at $p < 0.05$. Nodes represent OTUs and soil factors with different shapes, and OTUs are colored by genus-level taxonomy. *nosZ*: denitrifiers harbouring *nosZ* gene.

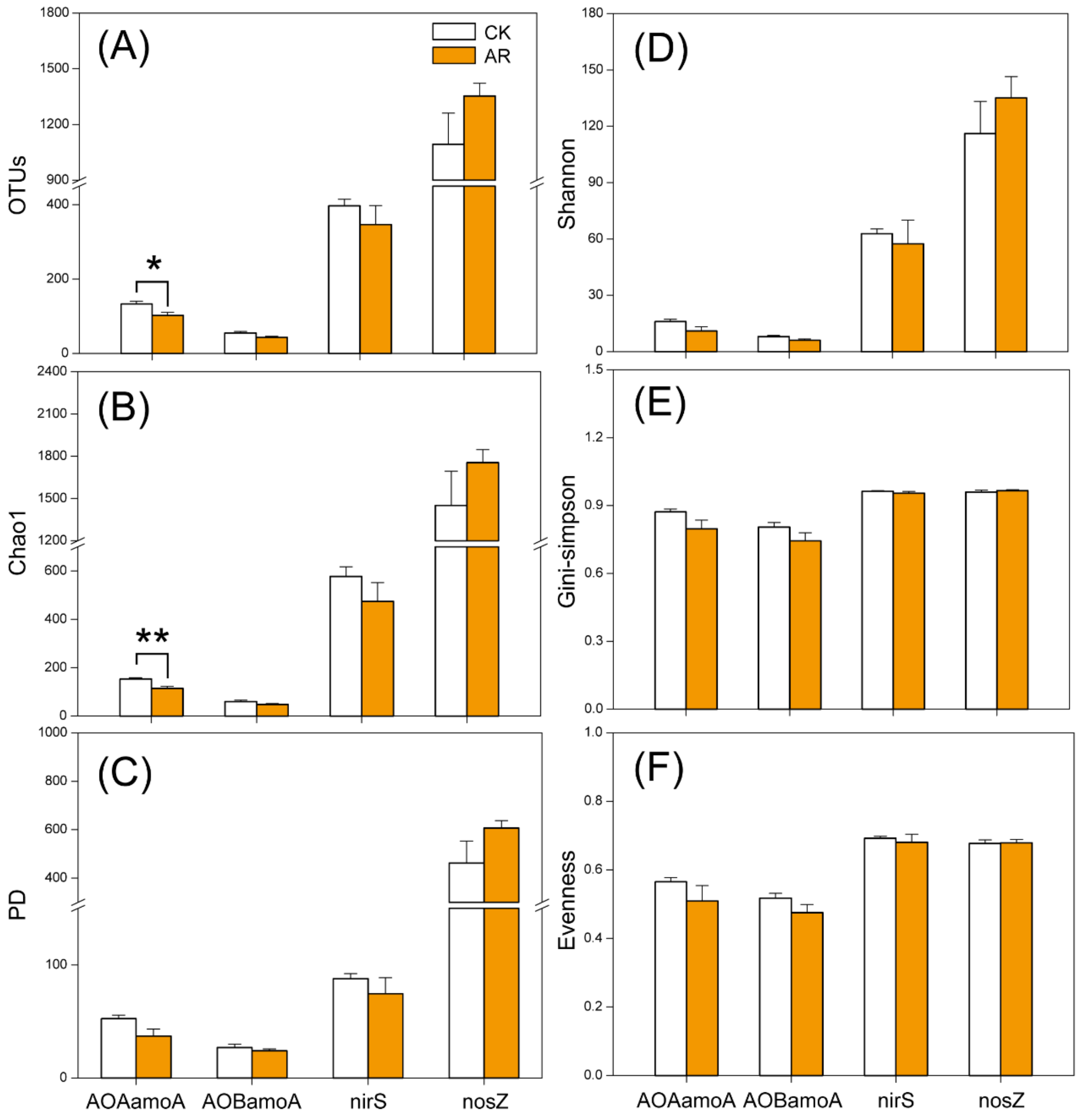

Figure S2. The microbial community richness and diversity indices of functional genes among treatments. OTUs: observed number of OTUs **(A)**; Chao1: Chao1 richness index **(B)**; PD: phylogenetic diversity index **(C)**; Shannon: Shannon diversity index **(D)**; Gini-Simpson: Gini-Simpson index **(E)**; Evenness: Shannon's evenness index **(F)**. CK: non-acid addition, AR: pH 4.0 acid rain. Data were presented as mean ± standard error ($n = 5$). ** $p < 0.01$, * $p < 0.05$.

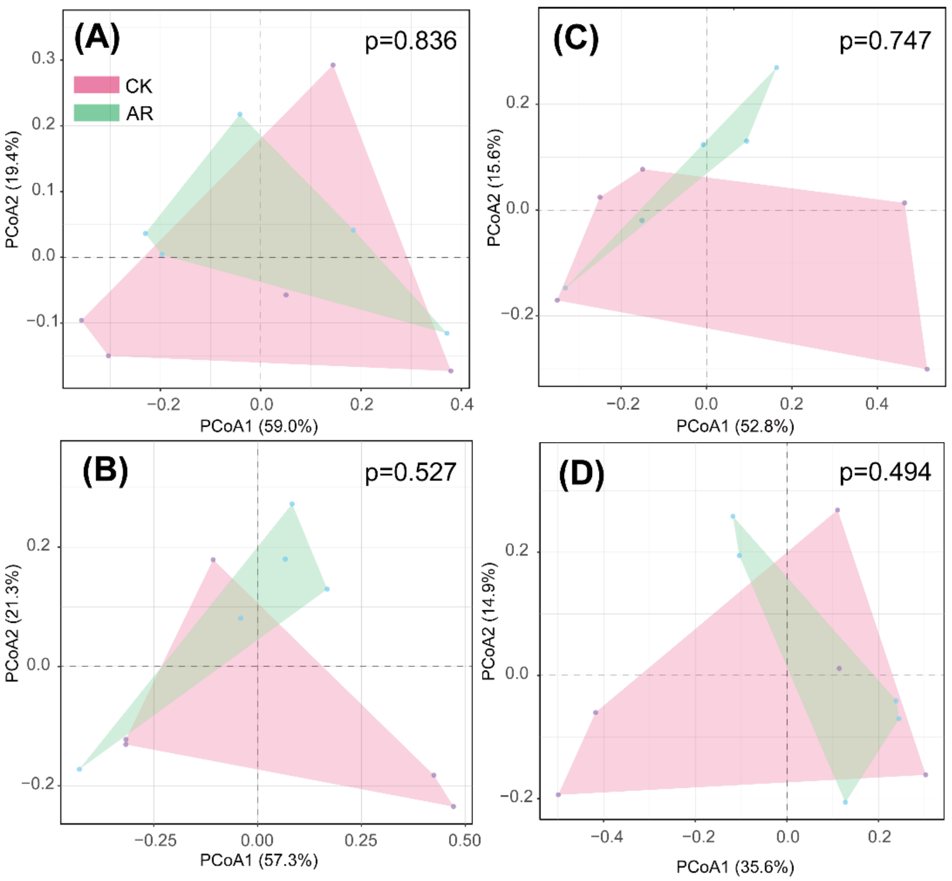

Figure S3. The principle coordinate analysis (PCoA) of the microbial communities structure of AOA **(A)**, AOB **(B)**, *nirS*-harboring denitrifiers **(C)** and *nosZ*-harboring denitrifiers **(D)**. CK: non-acid addition, AR: acid rain of pH 4.0. Five points in the same color block represent one treatment. The *p*-values refer to PERMANOVA results.

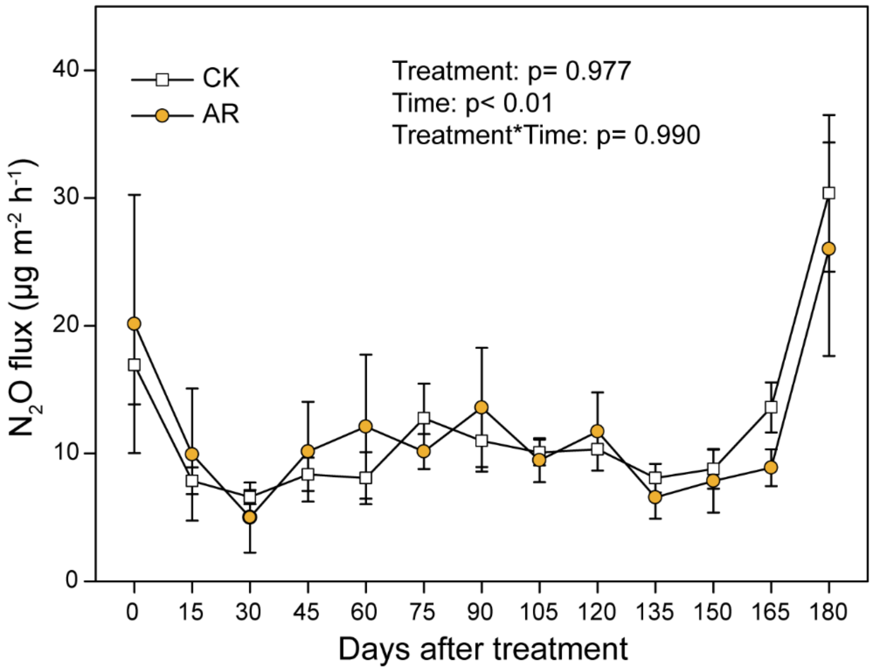

Figure S4. N_2O fluxes over sampling time. CK: non-acid addition, AR: pH 4.0 acid rain. Data were presented as mean ± standard error (n = 5).
